# Multiple signaling pathways of orexin receptors

**DOI:** 10.1186/2193-1801-4-S1-L17

**Published:** 2015-06-12

**Authors:** Jyrki Kukkonen

**Affiliations:** University of Helsinki, Finland

**Keywords:** Orexin, hypocretin, phospholipase

Orexin/hypocretin peptides are central for the regulation of the sleep/arousal states, and have, in addition, a role in the regulation of e.g. metabolism, addiction, stress response and pain gating. Orexin responses are mediated by G-protein-coupled OX1 and OX2 orexin receptors. Orexin signaling (in different cell types) is very versatile, ranging from excitation to induction of cell death. Orexin receptor coupling is promiscuous, engaging members of at least three different families of G-proteins, namely Gi, Gs and Gq, and some non-G-protein mediators as well. Preferred G-protein-coupling of the receptors appears different in different tissues, but the mechanism determining this are unknown. The primary signal transducers of orexin receptors very effectively activate phosholipase cascades, including PLA2, PLC and PLD, and also PLC-diacylglycerol lipase-mediated endocannabinoid generation. These cascades may play a significant role in the regulation of K+ and non-selective cation channels, which are the effectors for the orexin-mediated neuronal excitation. In some cell types, orexin receptor stimulation induces programmed cell death. Some different mechanisms for his have been proposed, but the picture is still incomplete. Orexin receptors have been a target for multiple drug discovery projects. Main focus has been on the antagonists, with insomnia as the indication. For agonist drugs there are some ongoing smaller academic and semi-academic projects. Use of orexin receptor agonists is suggested to be beneficial in narcolepsy (as peptide replacement therapy) and putatively in other sleep/wakefulness disturbances, metabolic disorders and cancer.

